# First retrospective studies with etiological confirmation of porcine transmissible gastroenteritis virus infection in Argentina

**DOI:** 10.1186/s12917-018-1615-9

**Published:** 2018-09-24

**Authors:** Pablo Enrique Piñeyro, Maria Inez Lozada, Laura Valeria Alarcón, Ramon Sanguinetti, Javier Alejandro Cappuccio, Estefanía Marisol Pérez, Fabio Vannucci, Alberto Armocida, Darin Michael Madson, Carlos Juan Perfumo, Maria Alejandra Quiroga

**Affiliations:** 1 Veterinary Diagnostic Laboratory, 1655 Veterinary Medicine, Iowa State University, 1850 Christensen Drive, Ames, IA 50011 USA; 2 Laboratorio de Patología Especial Veterinaria FCV-UNLP, Calle 60 y 118 S/N (1900), La Plata, Buenos Aires Argentina; 3 HIPRA Argentina, Saenz Peña R. Pte. Av 1110, Capital Federal, Argentina; 4 DILACOT-SENASA, Av A Fleming 1653, Martinez, Buenos Aires Argentina; 5 EEA Marcos Juaréz, INTA, CONICET, Ruta 12 km. 3 (2580), Marcos Juárez, Córdoba Argentina; 6 Veterinary Diagnostic Laboratory, University of Minnesota, 1333 Gortner Ave, St Paul, MN USA

**Keywords:** Porcine transmissible gastroenteritis virus, Diarrhea, Mortality, Piglets

## Abstract

**Background:**

In 2014, a notification of porcine transmissible gastroenteritis virus (TGEV) was made by the National Services of Animal Health of Argentina (SENASA) to the World Organization of Animal Health (OIE). The notification was based on a serological diagnosis in a small farm with a morbidity rate of 2.3% without enteric clinical signs. In order to determine if TGEV was circulating before the official report, a retrospective study on cases of neonatal diarrhea was performed. The selection criteria was a sudden increase in mortality in 1- to 21-day-old piglets with watery diarrhea that did not respond to antibiotics. Based on these criteria, three clinical cases were identified during 2010–2015.

**Results:**

All animals that were evaluated presented histological lesions consistent with enteric viral infection. The feces and ultrathin sections of intestine that were evaluated by electron microscopy confirmed the presence of round particles of approximately 80 nm in size and characterized by finely granular electrodense nucleoids consistent with complete particles of coronavirus. The presence of the TGEV antigen was confirmed by monoclonal specific immunohistochemistry, and final confirmation of a metabolically-active virus was performed by in situ hybridization to detect a TGE mRNA encoding spike protein. All sections evaluated in this case were negative for PEDV and rotavirus A.

**Conclusions:**

This is the first case series describing neonatal mortality with etiological confirmation of TGEV in Argentina. The clinical diagnosis of TGEV infections in endemic regions is challenging due to the epidemiological distribution and coinfection with other enteric pathogens that mask the clinical presentation.

**Electronic supplementary material:**

The online version of this article (10.1186/s12917-018-1615-9) contains supplementary material, which is available to authorized users.

## Background

There are five coronaviruses (CoVs) known to infect swine, and the clinical disease is mainly associated with neonatal diarrhea, but respiratory and neurological signs have also been reported [1–4]. Porcine transmissible gastroenteritis virus (TGEV) and porcine epidemic diarrhea virus (PEDV) belong to the *Coronaviridae* family, *Coronavirinae* subfamily, and genus *Alphacoronavirus* [5]. A new coronavirus genetically distinct from TGEV and PEDV, porcine deltacoronavirus (PDCoV) (genus *Deltacoronavirus*), has recently been associated with enteric disease in pigs [6]. Enteric porcine coronaviruses including TGEV, PEDV and PDCoV are characterized by acute diarrhea and anorexia with rapid dissemination in naïve populations. The severities of clinical diarrhea, vomiting, and anorexia can vary based on the age of the affected pigs [7, 8]. Without adequate passive lactogenic immunity, the mortality rate in neonatal piglets can reach up to 100% [1, 9–11]. Etiological diagnosis relies mainly on molecular tools like PCR and serology, as the clinical signs and enteric lesions associated with TGEV and PEDV are indistinguishable [8, 12, 13].

The epidemiology of TGEV is rather complex, and infection in neonates can arise from multiple sources. In addition to swine, it has be documented that cats, dogs, and foxes can host TGEV [14]. The virus can be shed in feces for approximately 18 months, and milk shedding from infected sows can result in vertical transmission. Historically, TGEV infection has followed a seasonal pattern, becoming more prevalent during winter months perhaps due to increased viral survival in colder temperatures and with less exposure to sunlight. TGEV is susceptible to most commercial disinfectants, but resistant to digestive bile and stable at pH 3 [1]. Only one TGEV genotype has been described, however differences in pathogenicity among strains has been reported in field outbreaks, although not confirmed by an experimental study [12].

Infection with TGEV has two different clinical presentations: epidemic and endemic. In epidemics, TGEV enters a naïve herd and all pig categories are affected, particularly piglets that are 1–2 weeks old. The duration of the clinical presentation is short, approximately 3 weeks, and in small, farrow-to-finish herd, the infection can be self-limiting [1, 14]. Endemic disease scenarios, those occurring after the epidemic phase, are observed in farms with incomplete AIAO management or in breeding farms that have a continuous flow of naïve gilts. In breeding herds, the varying levels of humoral and lactogenic immunity lowers piglet mortality, but may lengthen the course of the disease [1].

Since the first description provided in the United States [15], TGEV infections have been reported all over the world. In South America, it has been reported in Colombia [16], Venezuela [17], Bolivia, and is currently seen in Brazil [18]. In Argentina, an episode of high pre-weaning mortality related to *Isospora suis* infection alone or in association with an unknown enteric virus was reported in 1998 [19]. Further studies using negative stain electron microscopy demonstrate the presence of viral particles consistent with coronavirus in feces of pre-weaning diarrheic (34.4%) and post-weaning (10%) piglets [20]. A retrospective histopathological study performed on cases of neonatal diarrhea at our laboratory during 2013 showed that 29% of neonatal diarrhea cases had lesions consistent with viral enteritis [21]. In 2014, a notification of TGEV infection was reported by the National Services of Animal Health of Argentina (SENASA) to the World Organization of Animal Health (OIE). It was detected by serology in a small farm with an apparent morbidity rate of 2.3% without clinical signs [22]. This is an unusual presentation of TGEV infection and might be related to passed infection or interspecies transmission [1].

In order to clarify the situation prior to the first official report of TGEV infection in Argentina, a retrospective study was performed on cases suspected of TGEV-like disease recorded at the Laboratory of Special Veterinary Pathology at the College of Veterinary Sciences, La Plata University. Benchmarking analyses of epidemiological behavior and clinical histories were the criteria for herd selection. Etiological diagnosis was confirmed by electron microscopy (EM), immunohistochemistry (IHC), and in situ hybridization (ISH-RNA) of archived paraffin blocks.

## Results

### Clinical, pathological and etiological findings

#### Case 1

**Fig. 1:**
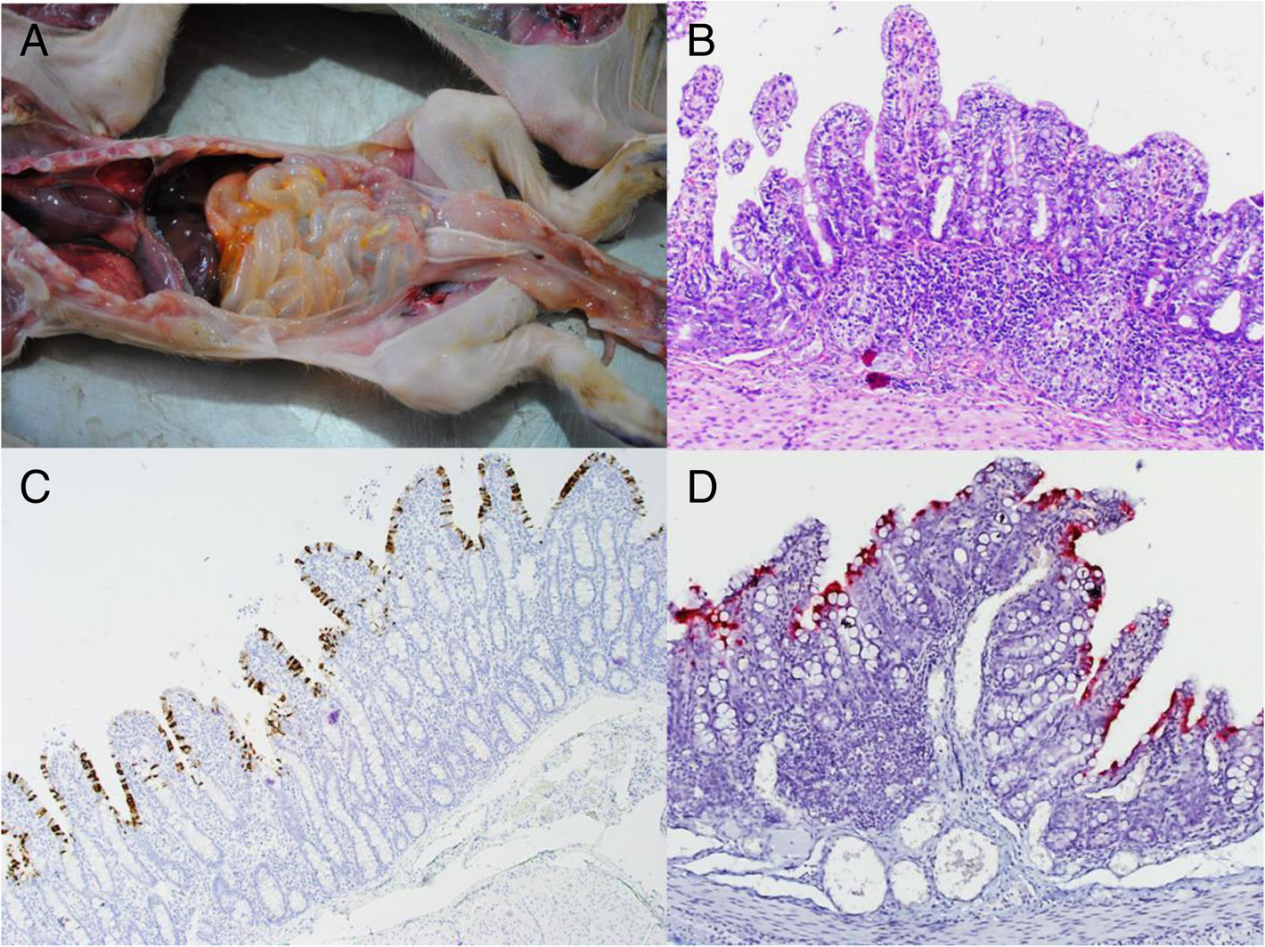
Gross and histological findings in neonatal piglets affected with TGEV detected in case 1. **a** shows thin small intestinal walls and loops distended by gas containing scant yellow watery digesta. **b** shows histological changes characterized by villous shortening, fusion and moderate submucosal edema. **c** and **d** present viral detection by IHC and ISH-RNA respectively. Note the severity of viral distribution affecting the entire length of the villi

Thirteen 1- to 7-day-old piglets with body weights ranging from 1 to 1.5 kg were submitted for pathological investigation. Pigs with clinical diarrhea were dirty, wet, had stained perinea, and showed moderate dehydration characterized by sunken eyes and diffusely pale mucosa. Their small intestinal walls were thin with unremarkable mesenteric lymphatic vessels, and were distended by gas or occasionally contained yellow watery digesta with a pH of 5–6 (Fig. 1a). Their stomachs were empty or contained floccules of undigested milk. No other macroscopic findings were observed. The histopathological evaluation showed a shortening of intestinal villi with a crypt-villous ratio of 1:2 (Fig. 1b), that villi were fused and lined by vacuolated cuboidal or attenuated epithelium, and that the lamina propria was expanded by moderate edema. Microscopic lesions were limited to the jejunum and ileum. Immunohistochemistry and ISH-RNA against TGEV showed strong staining in the epithelium of sections that presented minimal epithelial villous changes (Fig. 1c, d). Conversely, sections with the most severe epithelial damages were IHC negative. All sections evaluated in this case were negative for PEDV and rotavirus.

#### Case 2

**Fig. 2:**
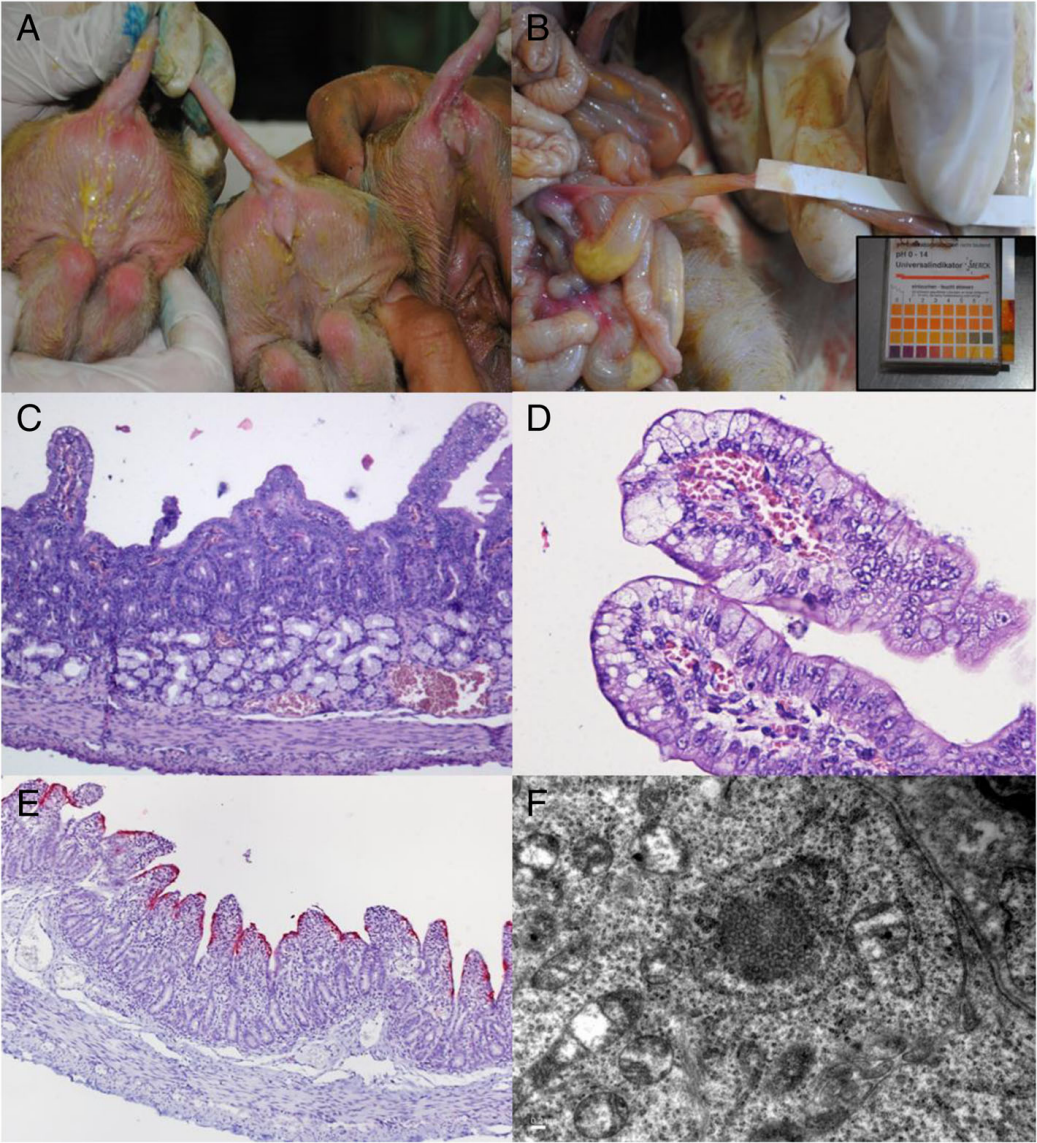
Gross and histological changes and viral detection in neonatal piglets affected with TGEV in case 2. Piglets presented with stool-stained perineum (**a**). Intestinal loops have thin walled, distended by gas and contain abundant yellow-watery diarrhea with 5–6 pH (**b**). Multiple sections of small intestine showed villous atrophy, blunting and fusion (**c**) with occasional cytoplasmic vacuoles (**d**). The presence of TGE mRNA confirmed in positive ISH-RNA enterocytes through multiple section of jejunum and ileum (**e**). **f** shows intracytoplasmic particles characterized by finely granular electrodense nucleoids with electron lucent centers compatible coronavirus

A gross evaluation of five piglets between 2 and 3 days old showed marked dehydration, wet and stool-stained perinea, and poor body conditions (1.15 kg average body weight) (Fig. 2a). The small intestine contained abundant yellow-watery diarrhea with a pH of 5–6 in two pigs and alkaline pH in three pigs (Fig. 2b). A histopathology examination of multiple sections of jejunum and ileum showed mild to moderate villous shortening and fusion (Fig. 2c). The villous enterocytes showed a marked cytoplasmic vacuolization (Fig. 2d). The lamina propria was minimally infiltrated by lymphocytes and plasma cells, and was expanded by edema. The superficial villous enterocytes in the ileum showed strong hybridization signals characterized by active-replicating TGEV (Fig. 2e). No microscopic lesions were seen in the sections of colon. All sections evaluated in this case were negative for PEDV and Rotavirus A. In the ultrathin sections, rounded particles measuring approximately 80 nm in diameter were located in the cytoplasm of the intestinal epithelial cells. The particles were characterized by finely granular electrodense nucleoids with electron lucent centers compatible with complete particles of coronavirus (Fig. 2f).

#### Case 3

**Fig. 3:**
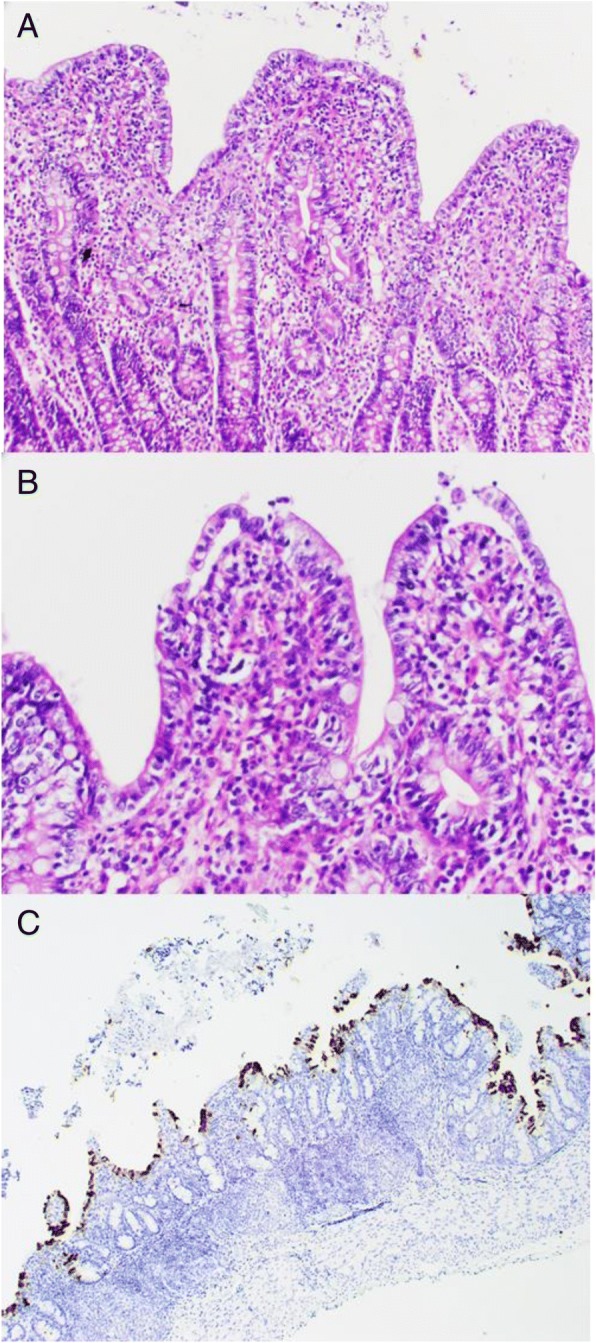
Histological changes and TGE-IHC detection small intestine in neonatal piglets affected with TGEV in case 3. **a** shows moderate villous atrophy and villi lined by low-cuboidal attenuated epithelium. There is also infiltration of the lamina propria by lymphocytes and plasma cells and minimal edema (**b**). Note the strong immunoreactivity against TGEV in superficial enterocitos (**c**)

Three neonatal piglets that died naturally presented with fecal stained perinea and were markedly dehydrated. The stomachs were empty, the small intestinal wall was thin/translucent, and scant yellow watery contents were sometimes apparent. The histopathology examination of the jejunum and ileum showed moderate villous fusion and shortening, and the villi were lined by low-cuboidal to flattened/attenuated epithelium (Fig. 3a). The lamina propria was infiltrated by numerous lymphocytes and plasma cells, was expanded by edema, and exhibited lymphangiectasia (Fig. 3b). The TGEV antigen was detected by IHC in multiple sections of the jejunum and ileum (Fig. 3c). No significant lesions were observed in the section of colon. All sections evaluated in this case were negative for PEDV and Rotavirus A.

## Discussion

The epidemiological and clinical presentations of outbreaks of neonatal mortality associated with enteritis and the detection of TGEV started in the gestation units. Both gilts and sows showed anorexia, diarrhea, and vomiting before enteric signs were observed in neonatal piglets. However, the prevalence of clinical signs in the breeding stock was low and no mortality was reported. When TGEV enters in a naïve herds, an epizootic form characterized by a 100% mortality of pre-weaning piglets due to diarrhea and dehydration is normally observed [1, 14]. Although in the present study, the farms had a high prevalence of diarrhea in suckling pigs, only farms A and C showed almost 100% neonatal mortality, while in farm B had approximately 20% neonatal mortality. The clinical presentation and epidemiological pattern observed in farm B resembled the TGE endemic form. Although no other etiological diagnosis was confirmed, the low mortality associated with TGEV that was observed in farm B might be the result of previous exposure to PRCV. PRCV infection can confer cross-protection against TGEV, reducing the enteric clinical signs and pre-weaning mortality [12]. Therefore, herds concomitantly infected with PRCV and TGEV develop less severe clinical signs, making the clinical differentiation from other enteric infections such as rotavirus or *E. coli* infections more challenging [1, 14]. Another potential reason for the low mortality rate due to TGE infection that was observed in farm B could be the intermittent viral exposure of the breeding stock that provided partial immunity to the neonatal piglets [14]. In all herds in this study, it was suspected that the virus entered the farms through the subclinically infected replacement animals that were not quarantined, or that the herds were exposed to the virus due to the proximity of a farm to a slaughterhouse (as seen in farm B).

Although the pre-weaning mortality rate in farm B was lower than that in farm A and C, the pre-weaning mortality was higher in this farm than the mortality rate seen in similar production systems due to other enteric causes such us *I suis* [19, 23], *C. perfringes* type A [24], or *C. difficile* [25]. According to a previous study, the pre-weaning mortality due to diarrhea should not exceed 20% [26]. Usually, in porcine CoVs infections, the course of the clinical disease is short and normally does not exceed 3–4 weeks [14] due to the establishment of a rapid herd immunity, as early as one week post-infection. However, in farm B the clinical presentation persisted for approximately 2 months. Potential reinfection due to poor husbandry and incomplete AIAO management are just few potential causes of viral persistence in the environment that can predispose reinfection of the farrowing units.

In clinically affected litters, most of the pigs are dirty, wet, and dehydrated, with diminished body weights. Diarrhea is watery, yellowish, and with an acidic smell from the presence of undigested milk [23]. The mortality rate is inversely correlated with the age of the piglets, reaching 100% in 2–7 day-old piglets. This predisposition is due to the slow replacement rate of villus epithelial cells (around 10 days) in neonatal piglets compared with the replacement rate of 3-week-old piglets (around 2–4 days) [14]. In this study, mortality varied from 20 to 100%.

The jejunum and ileum are the target segments of the small intestine for virus multiplication. However, TGEV infection is segmental, so multiple segments should be included for histopathology or etiological diagnoses in situ such as IHC or ISH-RNA. Due to the retrospective nature of this study, fixed tissue was used to confirm the presence of TGEV and the morphological changes consistent with viral infection in the intestinal mucosa. It is important to highlight that although TGEV was detected by different means in each farm, due to the segmental nature of the lesions and viral distribution, viral ARN or viral antigen was not detected in all of the intestinal segments that were evaluated. In piglets that are less than 2 weeks old, the reduction of villus length and the fusion of jejunum and proximal ileum are the main histological changes [27]. The normal villous/crypt length ratio is 7:1, however, after 24–48 h post-infection, the villous/crypt ratio can be reduced to 2:1 or 1:1 [27]. Since TGEV replicates in mature absorptive epithelial cells [28], a false negative diagnosis can be observed by IHC or ISH in specimens that display villous shortening. Few absorptive vacuolated cells are seen normally in the intestine, however, during TGEV infection, they are found in great numbers with a cuboidal shape [27]. In endemic TGEV infection, histopathological diagnosis is more difficult because only 25% of pigs display typical TGE lesions. In addition, immunofluorescence tests and IHC often fail in endemic farms due to a low number of enterocytes in the TGEV-infected because of partial protection conferred by colostral antibodies [23, 29]. Different diagnostic techniques have been used to detect TGEV infection such as IHC, ISH, electron microscopy, and immunoelectron microscopy and PCR [13]; however, histopathology remains the most useful tool for screening diagnosis [18]. In this study, although all cases were selected using clinical features and epidemiological information, the histological evaluation consistently showed lesions compatible with viral infection.

## Conclusions

The application of IHC and ISH-RNA on archived paraffin blocks from cases of neonatal diarrhea with high morbidity and mortality allowed retrospective identification of TGEV infection. Diagnosis of TGEV infection in endemic regions, such as in Argentina, is complicated due to the epidemiological distribution and clinical signs that might be masked with other enteric infections. Further studies are necessary to determine the true prevalence of this pathogen and the correlation with neonatal enteric cases observed in confined production systems.

## Methods

### Case selection and clinical history

Case selection was based on the clinical history reported by the farm managers and referring veterinarians. The selection criteria was a sudden increase in mortality that included, significant increment of more than 2 SD from average pre-weaning mortality and last for a period of a week. In addition reported mortality should be associated with the presence of watery diarrhea in 1- to 21-day-old piglets that did not respond to antibiotics. First screening of cases was done by histopathological evaluation, and only cases presenting features of viral enteritis with no other detected pathogen were included. Based on these criteria, three clinical cases were identified from 2010 to 2015.

#### Case 1

A 170-sow farrow-to-finishing herd located in Buenos Aires served as the first case. The farm produced its own replacement breeding stock, however, two months before the outbreak, gilts were introduced from a breeding company. The parity of the breeding stock was distributed as 40% gilts while the rest of the reproductive stock parity varied from 2 to 6. On September 2011, approximately 10% of the pregnant sows presented acute vomiting while 30% of the pregnant sows presented acute diarrhea. During the period when the sows showed gastro-enteric clinical signs, 2- to 4-day-old piglets presented vomiting (75–80%) and diarrhea (90%), and the mortality rate of suckling pigs reached 90%. The course of the disease in both breeder stock and piglets lasted for approximately three weeks.

#### Case 2

Case 2 involved a one-site herd of 350 sows with its own replacement gilts and the following parity distribution: 20% gilts, 40% parity 1–2, and 27.3% distributed amongst parity 3–5. Boars were purchased from a breeding company and were incorporated into the reproductive herd without quarantine. The farm was located within a few miles of a swine slaughterhouse in Buenos Aires. In February 2012, the pregnant sows showed anorexia (14–30%) and diarrhea (1%) associated with heat returns and abortions (3.3%). In the farrowing houses, approximately 100% of the lactating sows presented with anorexia. Pre-weaning mortality associated with the presence of diarrhea varied from 16.5% at the beginning of the outbreak to 27.9% 3 to 4 weeks after the initial clinical signs. An anatomopathological evaluation showed that 93.6% of the total pre-weaning mortality was due to diarrhea.

#### Case 3

A one-site herd of 400 sows was the subject of Case 3. In July 2013, two boars were located close to the gestation unit. A week later, gestation sows showed anorexia (16.8%) and diarrhea (5.3%). Thereafter, in the gilts, diarrhea was evident in the nursery (3–7%) and fattener (5–23%). Two-day-old piglets showed watery diarrhea (100%) with a mortality rate of 95%. Affected piglets died from severe dehydration within two days of the onset of clinical signs. The course of the disease lasted approximately two months with an overall pre-weaning mortality of 50% during that period.

### Pathological studies

At the onset of the outbreak, clinically affected suckling pigs were submitted for postmortem examination. Tissue samples from different organs, including multiple segments of small intestine (duodenum, jejunum, and ileum) and large intestine, were fixed in 10% buffered formalin, processed for routine histopathologic examination, and stained with hematoxylin and eosin.

### Etiological diagnosis in tissues and feces by immunohistochemistry, in situ hybridization, and electron microscopy

Immunohistochemestry IHC was carried-out briefly to differentiate TGEV [30] from PEDV [31]. Monoclonal antibodies against TGEV (OSU: #.14E3-3C) and PEDV (OSU 6C8) at dilutions of 1:8000 were used. Antigen retrieval was performed with humid heat and revealed with peroxidase (Novocastra^a^, Leica Biosystems, IL, USA). To rule out other potential viral enteritis, Rotavirus A was evaluated in all sections by IHC [32]. Rotavirus IHC was performed using a monoclonal antibody against Rotavirus A (Santa Cruz: sc-101363) at a 1:2000 dilution. Antigen retrieval was performed with Epitope Retrieval Solution 2 for 20 min, as programmed on Leica Bond III, and revealed with PowerVision Poly-HRP anti-Mouse (Leica PV 6113). All samples were tested in duplicate and sections were controlled appropriately for TGEV, PEDV and Rotavirus A with positive and negative controls (Additional file 1: Figure S1).

In situ hybridization ISH-RNA was developed through the RNAScope platform (Advanced Cell Diagnostics, Inc., CA), targeting the specific reverse complementary nucleotide sequence of the TGE viral mRNA (716–1859 region of spike gene, GenBank: KC609371.1). Therefore, positive hybridization signals represent a metabolically-active virus characterized by the TGE mRNA encoding spike protein. Unstained paraffin tissue sections were processed as previously described [33]. Briefly, tissues were deparaffinized and treated with hydrogen peroxide at room temperature for 10 min. The slides were hybridized using a hybridization buffer, and sequence amplifiers were added. The red colorimetric staining detected the TGE hybridization signal, and counterstaining occurred with hematoxylin.

Representative sections of small intestine were fixed in 2.5% glutaraldehyde and 2% paraformaldehyde in 0.1 M, pH 7.4 phosphate buffer (PBS) and post-fixed in 1% osmium tetroxide in PBS. After dehydration in an alcohol series, the fragments were embedded in epoxy resin, Quetol 812 (Nisshin EM Co., Ltd., Tokyo). Ultrathin sections were cut, double-stained with uranyl acetate-lead citrate, and observed under a JEM-1200EX (JEOL Co. Ltd., Tokyo).

## Additional file

Additional file 1: **Figure S1.** Panel of pathogens used as control for detection of TGEV by immunohistochemistry. Row one include immunostaining of TGEV clinical cases against TGEV, PEDV, and rotavirus specific antibodies. A moderate to severe immunostaining is observe only reacting against TGEV. In row two include positive controls for each pathogen detected by immunohistochemistry. Row three present section tested negative by PCR for TGEV, PEDV, and Rotavirus that were used as negative control of the immunohistochemistry techniques. (PPTX 3545 kb)

## Abbreviations

AIAO: All in all out
CoVs: Coronaviruses
EM: Electron microscopy
HRP: Horseradish peroxidase
IHC: Immunohistochemistry
ISH: In situ hybridization
mRNA: Messenger ribonucleic acid
OIE: World Organization of Animal health
PBS: Phosphate buffer saline
PCR: Polymerase chain reaction
PDCoV: Porcine deltacoronavirus
PEDV: Porcine epidemic diarrhea virus
SENASA: Argentina services of animal health
TGE: Porcine transmissible gastroenteritis
TGEV: Porcine transmissible gastroenteritis virus
