# Supplementary figures and images for: First retrospective studies with etiological confirmation of porcine transmissible gastroenteritis virus infection in Argentina
Source: BMC Vet Res. 2018 Sep 24;14:292. doi: 10.1186/s12917-018-1615-9 (PMC6154422; doi:10.1186/s12917-018-1615-9)

## Slide 1
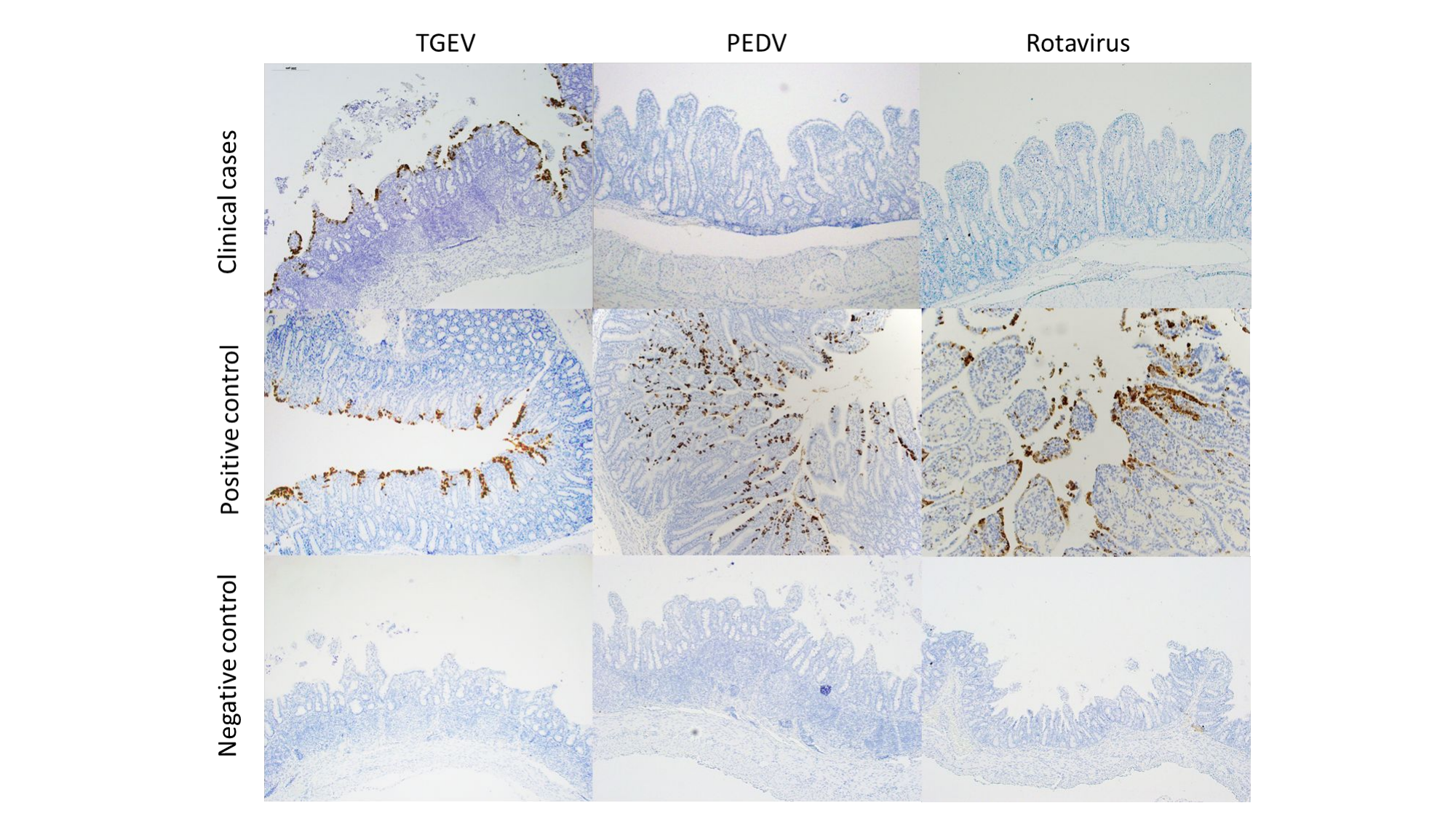

Supplement: Supplementary file 1 — Figure S1. Panel of pathogens used as control for detection of TGEV by immunohistochemistry. Row one include immunostaining of TGEV clinical cases against TGEV, PEDV, and rotavirus specific antibodies. A moderate to severe immunostaining is observe only reacting against TGEV. In row two include positive controls for each pathogen detected by immunohistochemistry. Row three present section tested negative by PCR for TGEV, PEDV, and Rotavirus that were used as negative control of the immunohistochemistry techniques. (PPTX 3545 kb) [file 12917_2018_1615_MOESM1_ESM.pptx]
